# Comprehensive dose-dependent hepatorenal protective effects of *Ziziphus nummularia* leaf extract against quinolphos-induced toxicity in Wistar rats

**DOI:** 10.3389/fphar.2026.1777534

**Published:** 2026-02-19

**Authors:** Ramya Rengaraj, Stalin Arumugam, Praveen Kumar Dharmaraj, Varadharajan Gokula, Aarthi Murugavel, Mohamed Saiyad Musthafa, Cristiana Roberta Multisanti, Caterina Faggio

**Affiliations:** 1 PG and Research Department of Zoology, National College (Autonomous) Affiliated to Bharathidasan University, Tiruchirappalli, Tamilnadu, India; 2 KIRND Institute of Research and Development Pvt Ltd., Tiruchirappalli, Tamil Nadu, India; 3 PG and Research Department of Zoology, Ethiraj College for Women (Autonomous), Affiliated to University of Madras, Chennai, Tamil Nadu, India; 4 Unit of Research in Radiation Biology and Environmental Radioactivity (URRBER), P.G and Research Department of Zoology, The New College (Autonomous), Affiliated to University of Madras, Chennai, Tamil Nadu, India; 5 Department of Veterinary Sciences, University of Messina, Messina, Italy; 6 Department of Chemical, Biological, Pharmaceutical, and Environmental Sciences, University of Messina, Messina, Italy; 7 Department of Ecosustainable Marine Biotechnology, Stazione Zoologica Anton Dohrn, Naples, Italy

**Keywords:** atrophy, hepatorenal toxicity, leukocyte infiltration, organ damage, organophosphate toxicity, quinolphos, *Ziziphus nummularia*

## Abstract

Quinolphos pose considerable hepatorenal toxicity risks, potentially necessitating the investigation of natural protective agents. This study assessed the pharmacological effects of *Z. nummularia* leaf extract in mitigating quinolphos-induced toxicity in male Wistar rats (n = 30) through comprehensive histopathological and behavioural evaluations. Animals were randomly divided into five groups: control, quinolphos-induced, vehicle control (300 mg/kg), and two treatment groups receiving *Z. nummularia* leaf extract at low dose (250 mg/kg) and high dose (500 mg/kg).The quinolphos-treated group displayed severe behavioural impairments, characterised by reduced locomotor activity, coarse coat texture, and substantial weight loss, with extensive histopathological damage in hepatic and renal tissues. Kidney sections exhibited significant glomerular cell debris and atrophy, abnormalities in the basement membrane, tubular degeneration, interstitial oedema, and tubular necrosis. The liver examination revealed significant changes in hepatocyte morphology, portal structures, leukocyte infiltration, sinusoidal congestion, and hepatocyte necrosis. The administration of *Z. nummularia* extract exhibited notable dose-dependent protective effects. The high-dose group demonstrated nearly complete histopathological recovery, with no detectable glomerular debris, tubular injury, or tubular necrosis in the kidneys, minimal hepatocyte changes, and total restoration of vascular integrity in liver tissue. Behavioural enhancements correlated with histopathological observations, reflecting a gradual restoration of appetite, activity levels, and overall clinical status. All treatment groups exhibited 100% survival during the experimental period. These findings reveal substantial evidence validating the pharmacological effects of *Z. nummularia* as a natural hepatoprotective and nephroprotective agent against organophosphate-induced organ damage.

## 1 Introduction

Pesticides are one of the most widely used tools in pest management globally, both in agriculture and in domestic settings (Kotia et al., 2025). In particular, organophosphates (OPs) and pyrethroids are among the most widely used classes in terms of effects, breadth of use and commercial availability (Bayrak et al., 2025; Cavallaro et al., 2023; Ramya et al., 2023; Roy et al., 2026; Tudi et al., 2021). However, the increasing intensification of industrial agriculture and the extensive use of chemical formulations, often associated with repeated treatments and mixtures of active ingredients, have increased the likelihood of release into the environment and accidental or chronic exposure of non-target organisms (Barathinivas et al., 2022; Hossinian et al., 2025; Sula et al., 2020; Stara et al., 2019; Vajargah et al., 2021).

Among these is quinolphos (O, O-diethyl O-quinoxalin-2-yl phosphorothioate), a versatile organophosphate insecticide, which has been widely used for crop protection due to its effectiveness against a diverse array of pests (Gupta et al., 2017). Its pervasive utilization has engendered considerable apprehensions regarding detrimental health implications for non-target organisms, including humans (Hernández et al., 2013; Ismail et al., 2026; Mostafalou and Abdollahi, 2016). In general OPs, including quinolphos, are known for their detrimental impact on liver and kidney function (Saha et al., 2025).

Pesticide exposure through occupational contact, aerial drift, and inadequate protective measures represents a major health concern for agricultural workers and nearby populations, contributing to hepatorenal damage and chronic diseases (Damalas and Koutroubas, 2016; Jayasumana et al., 2015; Lebov et al., 2015). Consequently, there is an urgent need to develop efficient detoxification approaches employing natural protective agents.

The liver and kidneys, key organs for xenobiotic biotransformation and elimination, are particularly vulnerable to pesticide-induced injury (Abdel-Daim et al., 2018; Jindal et al., 2024; Kalender et al., 2012; Mousaviyon et al., 2025). The liver serves as the primary site for OP metabolism, where bioactivation pathways can generate reactive intermediates and electrophilic metabolites that disrupt cellular homeostasis and promote hepatocellular damage (Abdel Moneim, 2016). In parallel, the kidneys, through their high perfusion rate and filtration function, may accumulate parent compounds and metabolites, making renal tissue especially prone to both acute and chronic pesticide-related nephrotoxicity (El-Demerdash, 2011; Mansour and Mossa, 2010). Quinolophos toxicity has been associated with excessive reactive oxygen species (ROS) generation and oxidative stress, leading to lipid peroxidation, mitochondrial dysfunction, and depletion or inhibition of endogenous antioxidant defences (Chromcova et al., 2015; Padmanabha et al., 2015). These interrelated events can trigger membrane destabilization, impaired energy metabolism, and activation of inflammatory cascades, ultimately culminating in structural and functional alterations such as cellular degeneration, inflammatory infiltration, necrosis, and progressive fibrotic remodeling in both hepatic and renal tissues (Padmanabha et al., 2015).

Due to the limitations and adverse effects of synthetic pharmacological agents, there is growing interest in researching natural alternatives that can alleviate the toxicity induced by contaminants (Akinwande et al., 2019; Multisanti et al., 2025a; Impellitteri et al., 2025). These include substances derived from plant sources, including plants (Multisanti et al., 2025b; Impellitteri et al., 2025). Plants belonging to the genus *Ziziphus* have been recognised in traditional medicine for their various pharmacological properties, such as antioxidant, anti-inflammatory, hepatoprotective, and nephroprotective effects (Pawlowska et al., 2009). The plant *Z. nummularia*, which is used in traditional medicine, has gained attention because of its alleged nephroprotective and hepatoprotective properties (Mesmar et al., 2022). *Z. nummularia*, referred to as wild jujube, is a xerophytic shrub indigenous to arid and semi-arid areas of Asia and Africa. Phytochemical analyses have revealed bioactive compounds in *Z. nummularia* leaves, such as flavonoids, saponins, alkaloids, tannins, and phenolic compounds, which collectively enhance its pharmacological potential (Mesmar et al., 2022). Ethnobotanical surveys document its traditional use in South Asian folk medicine for treating fever, inflammation, gastrointestinal disorders, and pain, with leaves and bark particularly valued for hepatoprotective properties (Khare, 2011; Abbasi et al., 2012).

Although various *Ziziphus* species have demonstrated hepatoprotective and nephroprotective effects, information on *Z. nummularia’s* protective effects against organophosphate-induced toxicity remains limited. Previous studies have primarily focused on biochemical parameters, with fewer incorporating comprehensive histopathological and behavioral assessments (Farkhondeh et al., 2024; Yu et al., 2021).

This study systematically investigates the hepatoprotective and nephroprotective properties of Z. nummularia leaf extract against quinalphos-induced toxicity in Wistar rats. Using an integrative approach combining standardized histopathological lesion scoring (0–3 scale) with behavioral evaluations, we aim to establish the dose-dependent protective effects of the extract and elucidate the relationship between microscopic tissue changes and functional outcomes.

## 2 Materials and methods

### 2.1 Plant material and extract preparation

*Ziziphus nummularia* (Burm.f.) Wight & Arn. [Rhamnaceae; Ziziphi nummulariae folium] were collected in January 2025 from Nallur, Thiruvarur district, Tamil Nadu, India (10.930317°N, 79.315222°E). The plant was authenticated by Fr. L. John Peter Arulanandam SJ, St. Joseph’s College, Tiruchirappalli. A voucher specimen (Accession No. SJCR.R001) was deposited at the Rapinet Herbarium and Centre for Molecular Systematics, St. Joseph’s College, Tiruchirappalli, Tamil Nadu, India (PIN 620002).

Fresh leaves were washed, shade-dried at room temperature, and pulverized. The powdered material (500 g) was extracted with 80% ethanol (1:10 w/v) by cold maceration for 72 h with occasional shaking, following established protocols for Ziziphus species (Mesmar et al., 2022; Abdallah et al., 2024). The macerate was filtered through Whatman No. 1 filter paper and concentrated under reduced pressure at 40 °C using a rotary evaporator to obtain a dark brown residue (yield: 14.2% w/w; drug-extract ratio 7:1). The dried extract was reconstituted in distilled water at 250 and 500 mg/kg body weight for oral administration. GC-MS analysis identified 48 phytochemical metabolites, with 3-methylsalicylic acid derivative (10.05%), cyclotrisiloxane hexamethyl (8.28%), and 2,3,4,6-tetrahydroisoquinolin-6-one derivative (7.46%) as major metabolites, confirmed using NIST and Wiley mass spectral libraries (≥80% similarity). Plant collection and processing followed Good Agricultural and Collection Practice guidelines (GACP) for medicinal plants (AHPA, 2006).

### 2.2 Experimental animals, housing, and ethical considerations

Thirty adult male Wistar albino rats with body weights ranging from 100 to 120 g were utilized in this investigation. The animals were housed in groups of five per cage across six polypropylene cages and maintained under standardized laboratory conditions, including a temperature of 25 °C ± 2 °C, relative humidity of 65%–75%, and a 12-h light/dark cycle (12:12 h). Rats were provided *ad libitum* access to water and a standard pelleted rat diet consisting of 60% maize, 20% soybean, 15% bran, 3% molasses, 1.5% mineral mix, 0.5% salt, and 0.2% vitamin premix, procured from the Animal Feed Manufacturing Unit, Ministry of Agriculture. The animals were acclimatized to the laboratory environment for a period of 2 weeks before the commencement of experimental procedures. All experimental protocols were conducted in strict accordance with the guidelines established by the Institutional Animal Ethics Committee (JJC/BC/AH/010/2025 Dated 17-05.2025) and adhered to the ethical principles outlined in the Declaration of Helsinki (1964) (Human Experimentation, 2025) and its subsequent amendments to ensure humane treatment of animals. All treatments were administered via gastric intubation on alternate days for 28 days. Animals were humanely euthanized via administration of sodium pentobarbital to ensure a painless and stress-free procedure, and liver and kidney tissues were immediately excised for histopathological examination.

### 2.3 Experimental design

A total of 30 adult Wistar rats were randomly divided into five groups (n = 6 per group) as follows: Group I served as the Control Group and received distilled water administered orally via gastric intubation. Group II served as the Toxicant-Induced Group and was administered quinalphos alone to induce hepato-renal toxicity without any treatment intervention. Group III served as the High Dose Treatment Group and received quinalphos along with a high dose of *Z. nummularia* extract (500 mg/kg body weight) to evaluate the protective effect at a higher dose. Group IV served as the Low Dose Treatment Group and received quinalphos along with a low dose of *Z. nummularia* extract (250 mg/kg body weight) to evaluate the protective effect at a lower dose. Group V served as the Vehicle Control Group and was administered *Z. nummularia* along with the distilled water (300 mg/kg body weight) to rule out any effects caused by the vehicle alone.

### 2.4 Behavioural study

The effects of quinalphos-induced toxicity and the protective potential of *Z. nummularia* leaf extract in rats were evaluated through behavioral observations (Angrand et al., 2021). The investigation was conducted in a controlled setting with uniform lighting, humidity, and temperature. Individual rats from each experimental group were monitored throughout the course of the treatment at predetermined intervals. Among the parameters assessed were the rats movements within the cage, which were used to measure locomotor activity, with exaggerated or reduced movement observed and documented as signs of hyperactivity or neurological dysfunction. The study also documented changes in posture, responsiveness, and attentiveness of the animals to external stimuli such as handling or noise.

### 2.5 Histopathological analysis

Histopathological examination was conducted following the techniques of Galigher and Kayloff as modified by Sarkar et al. (2005). The liver and kidneys were excised and promptly preserved in 10% buffered neutral formalin for 24 h. The specimens were rinsed in tap water, dehydrated in progressively higher concentrations of ethanol, treated with xylene, and embedded in paraffin wax (melting point of 50 °C–56 °C). Paraffin sections were prepared at a thickness of 6 μm utilising a rotary microtome (Model MR 60, Russian); the sections were subsequently stained with Harris haematoxylin and eosin. Observation was conducted utilising a light microscope (Zeiss Axiophot, Germany), and images were captured with an automatic photomicrographic camera.

## 3 Results

### 3.1 Behavioral observations and clinical features

During the experimental period, all treatment groups exhibited 100% survival, with no mortality observed in any of the five groups (n = 6 per group) (Table 1).

**TABLE 1:** Behavioural signs, survival of rats, active responses and clinical features observed in induced quinolphos and *Z. nummularia* leaves extract.

Group no.	Group description	N.	No. of death	Dose (mg/kg)	Clinical features observed
1	Control	6	0	–	Normal behaviour, alert, active
2	Induced	6	0	–	Decreased activity, rough coat, weight loss
3	High dose	6	0	500	Gradual recovery, improved appetite
4	Vehicle	6	0	300	Stable
5	Low dose	6	0	250	Reduced movement, mild fatigue, slight coat dullness

N = 6 animals per group (30 total). Groups 2–4 received quinalphos (10 mg/kg cumulative dose). Dose column indicates Z. nummularia extract concentration. Group 4 received vehicle (*Z. nummularia*). 100% survival observed across all groups.

The control group and vehicle group displayed typical behavioural patterns, characterised by alertness, active locomotion, and a robust appearance. The quinolphos-induced group that received no treatment exhibited pronounced behavioural and clinical abnormalities, such as reduced activity levels, coarse coat texture, and significant weight loss, indicative of systemic toxicity.

The high-dose *Z. nummularia* extract group (500 mg/kg) demonstrated a gradual recuperation from quinolphos-induced toxicity, accompanied by a progressive enhancement in appetite and overall clinical status. The low-dose treatment group (250 mg/kg) demonstrated diminished locomotor activity, mild fatigue, and slight coat dullness, indicating partial alleviation of quinolphos-induced toxicity but incomplete recovery at this dosage level. The behavioural and clinical observations aligned with the histopathological findings, supporting the dose-dependent protective effects of *Z. nummularia* leaf extract against quinolphos-induced organ toxicity.

### 3.2 Renal histopathological alterations

Histopathological analysis of renal tissues demonstrated significant structural impairment in the quinolphos-treated group relative to the control and vehicle-treated groups (Table 2).

**TABLE 2:** Kidney histopathological changes observed in induced Quinolphos and *Z. nummularia* leaves extract.

Parameter/Region	Control	Vehicle (300 mg/dL)	Quinolophos-induced	Low dose (250 mg/dL)	High dose (500 mg/dL)
Glomerular cell debris/atrophy	–	–	+++	+	-
Basement membrane wrinkling/thickening	–	–	+++	+	+
Proximal tubule	–	–	+++	+	-
Distal tubule	–	–	+++	+	-
Tubular degeneration	–	–	+ ++	+	+
Intestinal odema Tubular necrosis Cortico medullary junction	– _ _	– _ _	+++ +++ +++	++ ++ +	- _ _

Grading scale: – = no changes, + = mild, ++ = moderate, +++ = severe/extensive. Kidney sections stained with H&E and examined at ×100 magnification. Control group received distilled water; Vehicle group received Z. nummularia all other groups received quinalphos (10 mg/kg) with Z. nummularia extract at indicated doses.

**FIGURE 1:**
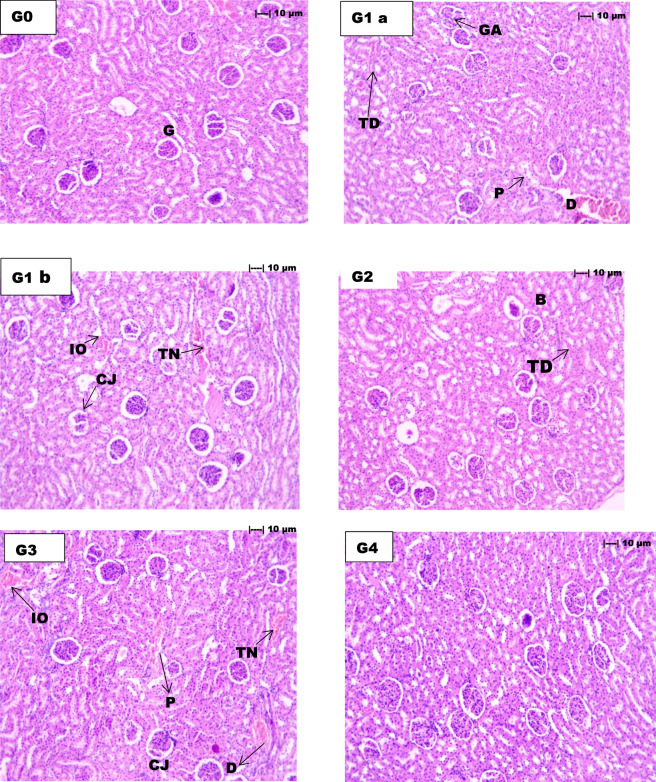
Histopathological evaluation of renal tissue sections (H&E staining, 100×) in Wistar rats showing quinolphos-induced nephrotoxicity and the ameliorative effects of *Z. nummularia* leaf extract at different dose levels. Control group **(G0)** exhibits normal glomerular (G) and tubular architecture. Quinolphos-induced groups **(G1a, G1b)** demonstrate severe pathological alterations including glomerular atrophy (GA), tubular degeneration affecting proximal (P) and distal (D) convoluted tubules, interstitial edema (IO), tubular necrosis (TN), and disruption at the corticomedullary junction (CJ). Treatment groups show dose-dependent recovery: High-dose *Z. nummularia* extract **(G2)**, 500 mg/kg displays preserved basement membrane (B) with mild tubular degeneration (TD); low-dose extract **(G3)**, 250 mg/kg shows moderate tubular necrosis and interstitial edema; vehicle control **(G4)**, 300 mg/kg presents normal histological features with no observable abnormalities.

The quinolphos-treated group (Figures 1G1a, G1b) displayed significant pathological alterations in various renal compartments, characterised by extensive glomerular cell debris and atrophy (+++), significant wrinkling and thickening of the basement membrane (+++), and severe damage to both proximal and distal tubules (+++). Tubular degeneration was significantly observed (+++), alongside severe interstitial oedema (+++), tubular necrosis (+++), and disruption of the corticomedullary junction (+++). No pathological changes were detected in the control and vehicle groups (300 mg/kg) (Figures 1G0, G4).

The administration of *Z*. *nummularia* leaf extract exhibited dose-dependent pharmacological effects against quinolphos-induced nephrotoxicity. The low-dose treatment group (250 mg/kg) (Figure 1G3) exhibited a mild presence (+) of glomerular cell debris, abnormalities in the basement membrane, damage to proximal and distal tubules, tubular degeneration, and disruption of the corticomedullary junction. Moderate interstitial oedema (++) and tubular necrosis (++) remained in this group. The high-dose treatment group (500 mg/kg) (Figures 1G2) demonstrated nearly total recovery, with no detectable glomerular cell debris, proximal and distal tubular injury, interstitial oedema, tubular necrosis, or corticomedullary junction disruption. Only minor alterations in the basement membrane (+) and tubular degeneration (+) were observed, signifying considerable nephroprotective effects at the elevated dosage.

### 3.3 Hepatic histopathological alterations

Microscopic examination of liver sections demonstrated significant hepatotoxic alterations in the quinolphos-treated group across various hepatic parameters (Table 3).

**TABLE 3:** Liver histopathological changes observed in induced quinolphos and *Z. nummularia* leaves extract.

Parameters/Region	Control	Quinolphos-induced	High dose (500 mg/kg)	Low dose (250 mg/kg)	Vehicle (300 mg/kg)
Hepatocyte/Hepatocyte cytoplasm	-	+++	+	++	-
Central vein	-	++	+	+	-
Hepatal portal vein/portal triad	-	+++	+	++	-
Leukocyte infiltration	-	+++	+	++	-
Cell lysis	-	++	+	+	-
Bile duct	-	++	+	+	-
Sinusoid congestion/digestion	-	+++	+	++	-
Vascular degeneration	-	++	-	+	-
Cellular tumefaction	-	++	+	+	-
Microvesicular/macrovesicular vacuolization	-	++	+	+	-
Focal leukocyte infiltration	-	++	+	+	-
Hepatocyte necrosis	-	+++	+	++	-

Grading scale: – = no changes, + = mild, ++ = moderate, +++ = severe/extensive. Liver sections stained with H&E and examined at ×100 magnification. Control group received distilled water; Vehicle group received Z. nummularia all other groups received quinalphos (10 mg/kg) with *Z. nummularia* extract at indicated doses.

Significant changes have been observed in hepatocyte morphology and cytoplasmic structure (+++), hepatic portal vein and portal triad architecture (+++), leukocyte infiltration (+++), sinusoidal congestion and degeneration (+++), and hepatocyte necrosis (+++). Moderate pathological alterations (++) were observed in the central vein, including cell lysis, bile duct abnormalities, vascular degeneration, cellular swelling, microvesicular and macrovesicular vacuolization, and focal leukocyte infiltration.

**FIGURE 2:**
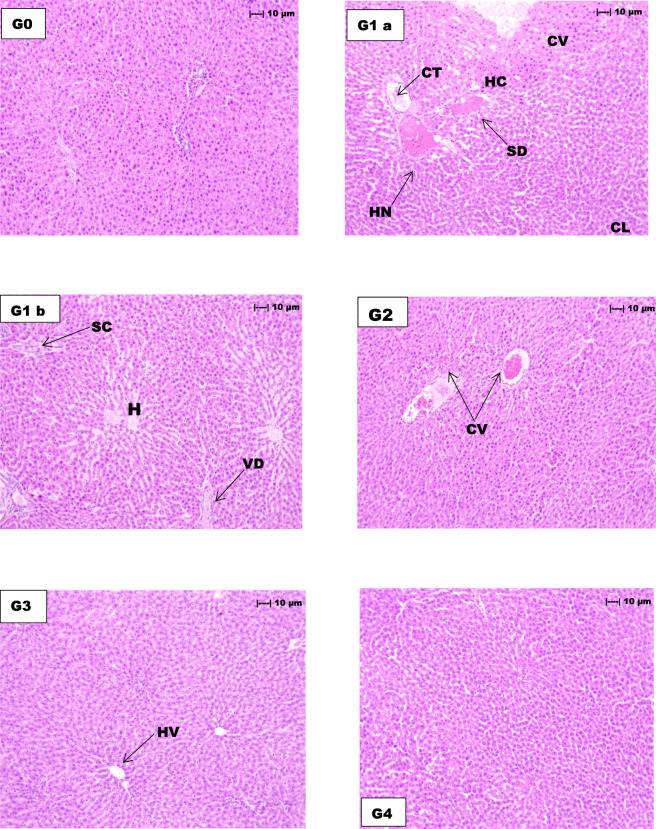
Histopathological evaluation of hepatic tissue sections (H&E staining, 100×) in Wistar rats demonstrating quinolphos-induced hepatotoxicity and the protective effects of *Z. nummularia* leaf extract at varying dose levels. Control group **(G0)** displays normal hepatic architecture with intact hepatocytes (H) and sinusoidal spaces. Quinolphos-induced groups **(G1a, G1b)** exhibit severe hepatotoxic manifestations including hepatocyte necrosis (HN), cellular tumefaction (CT), cytoplasmic vacuolation (CV) within hepatocyte cytoplasm (HC), sinusoidal dilation (SD), cell lysis (CL), vacuolar degeneration (VD), and sinusoidal congestion (SC). Treatment groups demonstrate variable recovery patterns: High-dose *Z. nummularia* extract **(G2)**, 500 mg/kg shows preserved centrolobular vein (CV) architecture with reduced pathological changes; low-dose extract **(G3)**, 250 mg/kg) reveals identifiable hepatic portal vein (HV) with moderate tissue restoration; vehicle control **(G4)**, 300 mg/kg displays normal hepatic architecture.

The control group and vehicle control group (Figures 2G0, 2G4) displayed no histopathological anomalies in any of the assessed parameters. The quinolphos-treated group (Figures 2G1a, G1b) displayed significant pathological alterations in hepatic architecture, characterised by extensive hepatocyte necrosis (+++), severe damage to hepatocyte cytoplasm (+++), and marked alterations in the hepatal portal vein and portal triad structures (+++). Leukocyte infiltration was significantly observed (+++), alongside severe sinusoidal congestion and dilation (+++). Additional hepatotoxic manifestations included moderate central vein congestion (++), cell lysis (++), bile duct alterations (++), vascular degeneration (++), cellular tumefaction (++), macrovesicular vacuolization (++), and focal leukocyte infiltration (++), collectively indicating extensive quinolphos-induced hepatocellular damage.

The administration of *Z. nummularia* leaf extract at both low (250 mg/kg) and high (500 mg/kg) (Figures 2G2) doses produced significant hepatoprotective effects. The low-dose group (Figures 2G3) demonstrated a moderate presence (++) of hepatocyte alterations, hepatic portal vein modifications, leukocyte infiltration, and hepatocyte necrosis, alongside a mild presence (+) of central vein changes, cell lysis, bile duct modifications, vascular degeneration, cellular tumefaction, vacuolization, and focal leukocyte infiltration. The high-dose treatment group exhibited enhanced protective effects, with only mild presence (+) observed across various parameters, including hepatocyte morphology, central vein, hepatic portal structures, leukocyte infiltration, cell lysis, bile duct, sinusoidal congestion, cellular tumefaction, vacuolization, focal leukocyte infiltration, and hepatocyte necrosis. Significantly, vascular degeneration was absent in the high-dose group, demonstrating considerable restoration of hepatic vascular integrity.

## 4 Discussion

The study examines the protective effects of *Z*. *nummularia* leaf extract against quinolphos-induced toxicity in Wistar rats, providing valuable insights into its potential pharmacological applications for alleviating pesticide-induced organ damage. The results of the study reveal significant changes in behavioural patterns and histopathological structures, supporting the protective function of the extract. During the experimental period, a significant disparity was observed in the behaviour of the control group compared to the quinolphos-exposed rats. The control group exhibited normal activity levels, characterised by alertness and active movement, whereas the quinolphos exposure group demonstrated notable behavioural abnormalities, including diminished locomotor activity, as indicated by a rough coat texture and substantial body weight loss. The noticeable reduction in activity and coarse coat texture suggests systemic toxicity linked to cholinesterase inhibition from organophosphate exposure (Salvi et al., 2003). The observed behavioural deficits correspond with other research indicating that exposure to agrochemicals, including organophosphates, causes systemic toxicity, leading to discernible behavioural abnormalities in rodent models (Badr, 2020). These behavioural alterations align with established effects of organophosphate pesticides that impair central nervous system function, leading to reduced motor skills and cognitive deficits (Chen et al., 2021). The decline in mobility and grooming within the quinolphos group corresponds with documented toxicological effects of organophosphate exposure (Badr, 2020).

The study also emphasised a dose-dependent response to the *Z. nummularia* extract. The behavioural recovery noted in the high-dose *Z. nummularia* extract group (500 mg/kg) indicates a substantial dose-dependent pharmacological potential, suggesting that this extract may mitigate the neurotoxic effects of quinolphos. The high-dose treatment group exhibited significant improvements, evidenced by recovery from anorexia and enhanced grooming behaviours, corroborating the hypothesis that elevated doses of natural extracts frequently augment protective effects against induced toxicity (You et al., 2025). The enhancement in appetite and overall clinical status in the high-dose treatment group signifies successful detoxification and reestablishment of physiological homeostasis, aligning with research that points out the hepatoprotective properties of diverse medicinal plant extracts (Olukanni et al., 2025). The phytochemical characteristics of the *Ziziphus* extract, which are known to contain bioactive compounds with potential antioxidant activity, may be partially responsible for the behavioural recovery seen when the extract was administered (Mesmar et al., 2022; Ray et al., 2024). This underscores the importance of *Z. nummularia* in traditional medicine, especially regarding its application as a neuroprotective agent (Boutlelis et al., 2025).

In the low-dose treatment group (250 mg/kg), partial symptom alleviation was noted; however, persistent indications of diminished locomotor activity and fatigue suggest that an increased dosage may be required for maximal effects. This corresponds with findings highlighting the importance of concentration in deploying plant extracts as antidotes against chemical-induced toxicity (Rongzhu et al., 2007). The observed behavioural enhancements correspond with changes in biochemical parameters, underscoring the protective properties of specific plant extracts against oxidative stress and inflammation (Liu et al., 2011).

Furthermore, the association between behavioural enhancement and histopathological observations indicates that *Z. nummularia* extract may operate on various levels to alleviate quinolphos-induced damage. *Z. nummularia* has become known for its bioactive compounds possessing antioxidant properties, potentially mitigating oxidative stress and inflammation caused by organophosphates (Dickey et al., 2007). This highlights the crucial function of natural extracts in safeguarding and revitalising impaired organ systems. The observed survival rate among treatment groups indicates that *Z. nummularia* administration may improve resistance to organophosphate toxicity. Moreover, specific *Ziziphus* species have demonstrated sedative, analgesic, anxiolytic, and anti-nociceptive effects, indicating their role in regulating central nervous system activity (Henneh et al., 2021; Kumar et al., 2023; Chung et al., 2025). The incorporation of traditional herbal remedies such as *Z. nummularia* into modern treatment protocols shows potential for mitigating organophosphate-induced toxicity, due to their natural composition and reduced side effects relative to synthetic pharmaceuticals (Mesmar et al., 2022; Imran et al., 2023).

The histopathological examinations indicate substantial insights into the structural damage caused to renal tissues by quinolphos exposure. The control and vehicle-treated groups exhibited no discernible pathological changes, in contrast to the quinolphos-treated group, which displayed significant alterations in multiple renal compartments. The results reveal significant glomerular cell debris, marked basement membrane irregularities, tubular injury, and interstitial oedema, suggesting considerable renal dysfunction due to the organophosphate toxin. These observations correspond with existing literature that records the nephrotoxic effects of organophosphates and their ability to induce acute kidney injuries characterised by glomerular and tubular disruptions, as noted in previous studies on various nephrotoxins (Sobolev et al., 2022).

The administration of *Z. nummularia* leaf extract exhibited significant nephroprotective effects that were dependent on dosage. The high-dose treatment group exhibited nearly complete recovery, reinforcing the hypothesis that phytochemical metabolites in *Z. nummularia* positively influence renal architecture and function. Prior research has validated these results, indicating that extracts from *Ziziphus* species exhibit antioxidant and anti-inflammatory properties that alleviate nephrotoxicity caused by various agents (Mesmar et al., 2022). The low-dose group demonstrated only minor histopathological alterations, further suggesting the extract’s potential to mitigate quinolphos-induced nephrotoxicity, likely due to its phytochemicalss, including flavonoids and saponins, recognised for their protective properties on renal tissues (Mesmar et al., 2021).

The noticeable improvement in kidney histopathology noted in the high-dose group corresponds with the increasing evidence indicating that natural extracts may serve as potent pharmacological agents against nephrotoxicity. Animal models administered different plant extracts have demonstrated a restoration of renal architecture and function, indicating their potential to mitigate the harmful effects induced by nephrotoxic agents (Jeshan et al., 2021). These findings suggest that *Z. nummularia* is a promising alternative for further pharmacological investigation and clinical use in renal protection against toxic environmental agents.

The microscopic examination of liver sections has provided essential insights into the hepatic histopathological changes caused by quinolphos. The studies indicate significant hepatotoxic alterations accompanied by profound changes in multiple hepatic parameters. The quinolphos-induced group exhibited significant abnormalities, including pronounced alterations in hepatocyte morphology, disturbances in the hepatic portal veins and triad structures, extensive leukocyte infiltration, sinusoidal congestion, and hepatocyte necrosis, all assessed with considerable severity. Furthermore, moderate pathological alterations were noted in additional parameters, encompassing changes in central vein structure, cellular lysis, bile duct pathology, vascular degeneration, cellular swelling, vacuolization, and localised leukocyte infiltration. The control group and vehicle group exhibited no histopathological abnormalities, highlighting the harmful effects of quinolphos.

The administration of *Z. nummularia* leaf extract exhibited substantial hepatoprotective effects at both low (250 mg/kg) and high doses (500 mg/kg). In the low dose group, moderate hepatocyte alterations, hepatic portal vein changes, and leukocyte infiltration were evident, indicating persistent hepatotoxicity. However, even in this group, improvements were observed in a number of evaluated parameters, such as hepatocyte necrosis, which was noticeably less severe than in the quinolphos group. The elevated dosage demonstrated enhanced protective effects, evidenced by a significant decrease in hepatocyte alterations and total resolution of vascular degeneration, thereby indicating a considerable restoration of hepatic vascular integrity, suggesting the intricate role of phytochemicals in mitigating chemically induced liver pathology (Mesmar et al., 2022).

This synergy indicates that *Z. nummularia* contains bioactive compounds that can mitigate oxidative stress and inflammation associated with hepatotoxicity, thereby supporting its traditional application in liver disorders. Multiple studies on *Z. nummularia* highlight its abundant phytochemical composition, encompassing alkaloids and flavonoids, which enhance its pharmacological advantages (Abdulrahman et al., 2022; Mesmar et al., 2022).

The present study confirmed our hypothesis that Z. nummularia leaf extract provides dose-dependent protection against quinalphos-induced hepato-renal toxicity. This hypothesis was substantiated by dose-dependent behavioral recovery (500 mg/kg > 250 mg/kg), near-complete histopathological resolution at high dose versus partial protection at low dose. The vehicle control exhibited no adverse effects, validating the non-toxic nature of the extract.

## 5 Conclusion

This study confirms our hypothesis that *Z. nummularia* leaf extract provides dose-dependent protection against quinalphos-induced hepato-renal toxicity. The integrative method that incorporates behavioural monitoring with comprehensive histopathological grading effectively revealed that quinalphos exposure causes significant multi-organ damage, characterised by prominent nephrotoxic and hepatotoxic changes, such as glomerular atrophy, tubular degeneration, interstitial oedema, hepatocyte necrosis, and sinusoidal congestion. The high-dose *Z. nummularia* treatment (500 mg/kg) resulted in nearly complete histopathological recovery in renal and hepatic tissues, with complete disappearance of most pathological parameters and restoration of vascular integrity, while the low-dose treatment (250 mg/kg) provided partial protection. The vehicle control group exhibited no adverse effects, confirming the safety profile of Z. nummularia extract. The identified protective mechanisms probably relate to the abundant phytochemical composition of *Z. nummularia*, especially flavonoids, saponins, and phenolic compounds with antioxidant and anti-inflammatory attributes that mitigate organophosphate-induced oxidative stress. The relationship between behavioural recovery and histopathological restoration indicates the systemic protective effects of the extract. These findings establish *Z. nummularia* as an appropriate option for development as a natural prophylactic or pharmacological agent for populations at risk of organophosphate exposure, particularly agricultural workers in areas with high pesticide usage, and necessitate further mechanistic investigations and clinical translation studies.

## Data Availability

The original contributions presented in the study are included in the article/supplementary material, further inquiries can be directed to the corresponding author.
